# 

**Published:** 1888-05

**Authors:** 


					﻿NOTICE.
JU articles for publication, books for review, business communications, etc.,
must be sent to Editors, 1123 Spruce Street, Philadelphia.
Subscribers will please notice that this Journal will be sent until arrears
are paid and it is ordered discontinued.
The best' is always the cheapest; but the cheapest is hot
always the best. Just here THE HOMOEOPATHIC PHY-
SICIAN contradicts all previous experience in being both
the cheapest and the best. See that your friends know this ! !
WANTED.
The following back numbers 6f .The Homoeopathic Physician are wanted -
twenty-fivecents will be paid, for each number received in . good condition.
Please look over your odd journals and see if you can help us. Write your
name on the wrapper. Volume eight will be sent in exchange for either
volume one, two, three, or six.
* Address The Homoeopathic Physician,
1123 Spruce Street, Philadelphia.
Volume First: Febuary, April, and June. .
Volume Sixth • January and November. :
				

